# The effectiveness of nifedipine/indomethacin combination therapy and nifedipine monotherapy for postponing preterm birth (25–34 weeks of gestation) in Sudanese women: a randomized clinical trial study protocol

**DOI:** 10.1186/s12884-021-03951-x

**Published:** 2021-06-29

**Authors:** Mohammed H. Ibrahim, Tahani Elfaki, Elhassan M. Elhassan, Somia K. Abdelrahiem, Ishag Adam

**Affiliations:** 1 Department of Pharmacology and Toxicology, Unaizah College of Pharmacy, Qassim University, Unaizah City, Qassim Region 51911 Saudi Arabia; 2 Department of Obstetrics and Gynecology, University of Gezira, Medani, Sudan; 3 Department of Obstetrics and Gynecology, Unaizah College of Medicine and Medical Sciences, Qassim University, Unaizah, Kingdom of Saudi Arabia

**Keywords:** Preterm labor, Tocolytics, Nifedipine, Indomethacin, Control trial

## Abstract

**Background:**

Preterm birth is the most common cause of neonatal morbidity and mortality. Tocolytics are considered a standard treatment for women with threatened preterm delivery to allow time for maternal steroid administration and transfer to referral centers with neonatal intensive care units. However, there is controversy about the best tocolytic therapy to be considered as the first choice. The aim of this study is to compare the tocolytic effectiveness and tolerability of combination therapy with nifedipine and indomethacin versus nifedipine monotherapy among Sudanese women with preterm labor (PTL) as well as to compare the possible neonatal outcomes associated with each drug.

**Methods/design:**

This is a randomized controlled clinical trial to be conducted in the Medani Maternity Hospital, Sudan. Women aged 18–40 years that are diagnosed with preterm labor and have a gestational age between 25 and 34 weeks will be eligible to participate in this trial. The diagnosis of threatened PTL is defined as persistent uterine contractions “(four contractions every 20 min or eight contractions every 60 min)” with cervical changes “(cervical effacement ≤80% or cervical dilatation >two cm)”. Patients will be eligible regardless of the presentation of the fetus. It will be randomly decided whether participants receive nifedipine/indomethacin combination therapy or nifedipine monotherapy. The primary outcome is the number of women who do not deliver and do not need alternative tocolytic drug (terbutaline). The secondary outcome is an estimated association with neonatal morbidity and mortality. The sample size will be 117 subjects in each arm of the study, according to a type I error of 0.05 and a study power of 80%.

**Discussion:**

We expect higher effectiveness of the combination indomethacin/nifedipine tocolytic therapy compared with nifedipine monotherapy. We plan to suggest this combination therapy as the best option for postponing PTL.

**Trial registration:**

Clinical trial registration: PACTR202004681537890, date of registration: March 8, 2020.

**Supplementary Information:**

The online version contains supplementary material available at 10.1186/s12884-021-03951-x.

## Background

Preterm labor (PTL) can be described as the presence of regular uterine contractions that result in cervical dilatation prior to the 37th week of pregnancy and is one of the leading causes of death and serious adverse events in newborns [1, 2]. PTL is associated with 50–75% of neonatal mortality and 50% of neonatal morbidity worldwide [3, 4]. PTL is also associated with economic burden due to the high requirements of neonatal intensive care units and socioeconomic disadvantages to the affected patients related to disruptive life events [5, 6]. PTL occurs in approximately 4–15% of all pregnancies, and these percentages have increased in recent years [5, 7]. The rates of PTL vary according to countries and regions; they are approximately 5% in some northern European countries but over 15% in some countries of sub-Saharan Africa and Asia [8]. In Sudan, preterm births occur in 3.8% of all pregnancies, and the majority (81.0%) of these preterm births are spontaneous [8, 9].

PTL-associated morbidity and mortality rates are inversely related to gestational age at delivery. Although 50% of PTL occurs after gestational week 35, the associated morbidity and mortality occurs before this time in 99% of cases [10]. Using tocolytics in women as labor-inhibiting agents could postpone delivery by inducing myometrium relaxation [11]. It has been shown that survival rate increases by 3% for every 24 h of labor delay, which allows for further neonatal rescue intervention and the administration of alternative treatments, such as antenatal corticosteroids, during the extended period until delivery [12, 13]. Several tocolytic classes are commonly used, including ritodrine hydrochloride, prostaglandin antagonists indomethacin and ketorolac, the β-adrenoceptor agonist ritodrine, magnesium sulfate, the oxytocin receptor antagonist atosiban, the calcium channel-blocking agent nifedipine, and others. These medications tend to be used due to their unique mechanisms of action. However, each one has its own profile, which includes adverse effects and methods of administration [14]. There are still controversies regarding their effectiveness and associated adverse events. A randomized clinical trial that investigated the effect of nifedipine versus placebo in the treatment of preterm contractions reported that nifedipine was more efficient compared with the placebo [15]. It has been suggested that combination therapy could be a viable option for the prevention of PTL [16]. A previous trial has shown promising results regarding the superiority of nifedipine and indomethacin as a combined therapy for PTL [17]. However, there was no significant difference in terms of efficacy and safety profile when three tocolytic agents (indomethacin, nifedipine and magnesium) were evaluated for the treatment of PTL [18, 19]. Importantly, none of these studies were conducted in Africa.

The aim of this study is to compare the tocolytic effectiveness and tolerability of a combination therapy consisting of nifedipine and indomethacin versus nifedipine monotherapy among Sudanese pregnant women with PTL as well as to compare the possible neonatal outcomes associated with each drug.

### Aims of the study

The primary objective of this study is to record the number of women at risk of PTL with a gestational age between 25 and 34 weeks who do not deliver and do not need alternative tocolytic drug (for 48 h) of the combined tocolytic agents nifedipine and indomethacin or the nifedipine monotherapy in delaying delivery for 48 h in women. The secondary objective is to compare the effectiveness of the two mentioned therapies in regard to improvements in neonatal outcomes among the same study groups. The outcomes include neonatal mortality and morbidity.

(SPIRIT Checklist Statement) Additional file 1.

**Table 1:** Study timetable and activities

Phase	Duration	Activities
**Phase 1**	**6 months**	1- Preparation of the study team
		2- Preparation of the study drugs
		3- Design of the study forms including patient database and inform consent
		4- Trial approval by the required organization or body
		5- Task distribution and training
**Phase 1**	**12 months**	1- Patient selection
		2- Randomization
		3- Administration of the trial treatment
		4- Patient evaluation (pre−/post-trial)
		5- Assessment of the target outcomes
		6- Evaluation of complications or adverse reaction, if any
		7- Analysis

## Methods/design

### Study type

This study will be a randomized controlled clinical trial.

#### Setting

Medani Tertiary Maternal Hospital in central Sudan.

### Patients

1. Inclusion criteria: women aged 18–40 years with a viable fetus diagnosed with threatened PTL and whose gestational age is between 25 and 34 weeks will be eligible for participation in this trial. A diagnosis of threatened PTL will be defined as persistent uterine contractions “(four contractions every 20 min or eight every 60 min)” with cervical changes “(cervical effacement ≤80% or cervical dilatation > 2 cm) [20]. Patients are eligible independent of the presentation of the fetus.
2. Exclusion criteria: Women who have had multiple pregnancies; any condition considered as a contraindication for tocolysis, e.g., hypertension or the use of anti-hypertensive medication, ruptured membranes, signs of fetal distress and/or intrauterine infection; a maternal temperature > 38 °C; severe vaginal bleeding; myocardial infarction within 3 months; unstable angina pectoris; cervical dilatation of more than 5 cm; cerclage; suspicion of neonatal chromosomal or structural anomalies; tocolytic treatment for more than 6 h prior to arriving in the participating hospital; and an unwillingness to participate in this study.

### Procedures, randomization, recruitment, and collection of study data

**Fig. 1:**
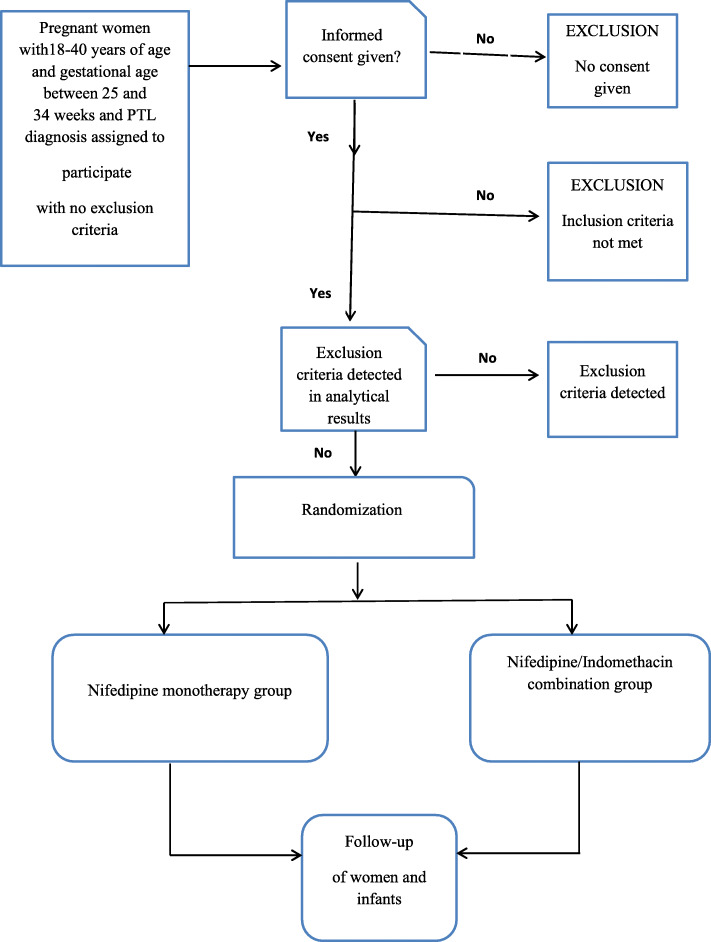
Patient recruitment flow chart

The study will be a randomized controlled trial conducted within the obstetric unit of a teaching hospital. All eligible patients to be admitted will be asked for informed consent. They will participate unless they do not offer permission, or if they meet any of the exclusion criteria (Fig. 1). After counseling, patients will be asked to read and sign a written informed consent form. After signing informed consent, the baseline demographics and obstetric and medical history of the patient will be entered into a specific database for the study. Body mass index will be calculated (BMI) from the measured weight and height. Randomized allocation using a computer will be carried out through numbers, and sealed opaque envelopes will be in blocks (8) for indomethacin/nifedipine combination therapy or nifedipine monotherapy. Each envelope which will contain an assignment for a single patient. The epidemiologist (IA) will assign the number, and these numbers will be used for the enrollment; we will then enroll the participants.

**Table 2:**
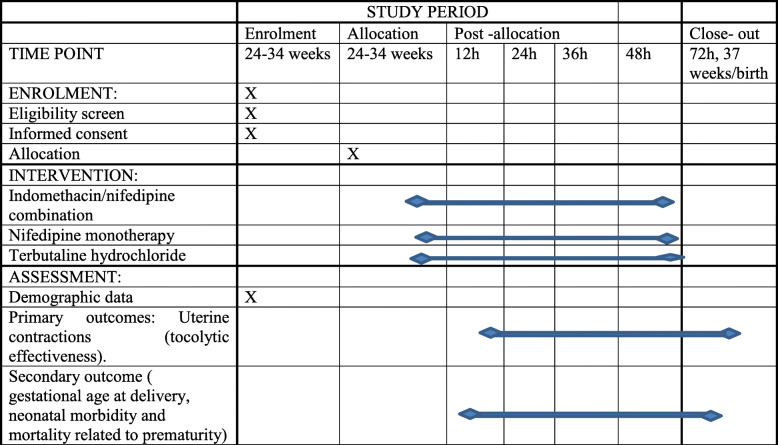
Schedule of enrolment, interventions, and assessments during the clinical trial

### Sample size

Each study group will consist of 117 women, a number calculated based on a previous study [17]. We expect the proportion of successful outcomes (the number of women who do not deliver and who do not need alternative treatment for 48 h) in nifedipine monotherapy to be 72.0%, and we expect it to be 89.0% in the combination group. This sample size (117 in each arm) was necessary to achieve a 95% confidence level with a 5% margin of error and 80% power.

### Blinding

Blinding may prove costly, logistically challenging, and technically difficult in our setting. Therefore, we opt to carry out an open randomized controlled trial.

### Interventions

Patients will be allocated to the indomethacin/nifedipine combination group or the nifedipine monotherapy group for 48 h. In the nifedipine group, the patients will receive 20 mg oral nifedipine. If contraction inhibition occurs for 2 h, the patients will continue to receive 20 mg of oral nifedipine every 4 h for 48 h, and the maximum dose will not exceed 180 mg per day. In the combination group, the patient will be administered nifedipine orally, as in the monotherapy group, and 100 mg indomethacin rectally. In the case of contraction inhibition after 2 h, the patients will receive 25 mg of oral indomethacin every 4 h. The maximum daily dosage of indomethacin will not exceed 200 mg/day, and the maximum duration will be 48 h. Terbutaline hydrochloride (the second-line drug) will be used in those cases (in both arms) of unsuccessful treatment and if there is no contraindication for terbutaline. Terbutaline hydrochloride will be used by intravenous drip, adjusted for its effectiveness in inhibiting uterine contractions [21]. Four doses of 6 mg dexamethasone will be given intramuscularly every 12 h [22].

### Outcome measurements

#### Primary outcomes

The monitoring of uterine contractions will be performed continuously by an external tocodynamometer for 2–4 h after the first administration of the study’s medications followed by two to three times per day for 30–60 min. The primary outcome will be an estimation of the tocolytic effectiveness and safety profile. The effectiveness will be assessed in terms of the proportion of women who do not deliver and who do not need alternate tocolytic medication (terbutaline) within 48 h after the first administration of the study drug. Safety assessment will be performed by assessing maternal-associated adverse effects that include maternal tachycardia (i.e., a heart rate above 120 beats per minute), hypotension (i.e., systolic blood pressure less than 90 mmHg), and other symptoms, such as palpitations, headache, nausea, vomiting, itching, and rash.

#### Secondary outcomes

Secondary outcomes will be the number of women with preterm birth before 37 weeks, women with preterm birth before 34 weeks, days until delivery, gestational age at delivery, neonatal morbidity and mortality related to prematurity (e.g., lung disease, severe intraventricular hemorrhage, periventricular leukomalacia, sepsis, necrotizing enterocolitis), and gestational age at delivery. These outcomes will be assessed by the neonatologist (with suitable/optimum investigations) until discharge from the hospital or neonatal death.

### Follow-up of women and infants

The delivery details, pregnancy and postpartum assessments for both mothers and neonates will be recorded in a hospital-based case follow-up form. In addition, all details of neonatal admission will be recorded.

### Analysis

Statistical analysis will be performed by using the SPSS version 22.0 (IBM) program (intention-to-treat analysis). In the evaluation of the study data, descriptive statistical methods (mean, standard deviation, minimum, maximum, median, frequency, and ratio) will be used. Student’s *t*-test will be used to compare quantitative data for normally distributed data, and if the distribution is not consistent with a normal distribution, the Mann–Whitney *U* test will be used to compare the variables between women in each arm of the study. Pearson’s chi-squared and Fisher’s exact tests will be used for qualitative data comparisons. Number needed to treat (NNT) for benefits and number needed to harm (NNH) for adverse outcomes with their 95% confidence outcomes will be calculated. A *p*-value> 0.05 will be considered statistically significant.

The groups (nifedipine/indomethacin combination group or nifedipine monotherapy group) will be analyzed as group A and group B, and confidentiality will be preserved. The identification of groups will be revealed only after the analysis is finished with the tables ready.

## Discussion

We are expecting a higher effectiveness of the combination indomethacin/nifedipine tocolytic therapy compared with nifedipine monotherapy. Among the various tocolytics, nifedipine seems to be the most appropriate therapy for PTL [15]. However, it has been suggested that combination therapy is a good option for postponing of PTL [16]. Our findings will be supported by the recent findings of Kashanian et al. who enrolled 150 participants; these investigators reported that 89.4% of the combination-therapy patients compared to 72.0% of the patients in either a nifedipine or indomethacin monotherapy group had inhibition of uterine contractions for 48 h. Accordingly, the investigators indicated combination therapy be employed for inhibiting preterm labor, delaying delivery, and prolonging the duration of pregnancy [17].

### Limitations

It is desirable to have a blinded clinical trial in this situation; however, this might be difficult to conduct in our setting. There is an expected degree of bias due to the lack of blinding in this protocol and this might affect the results of the trial. Some inflammatory markers will not be assessed and this could be one of the limitations.

## Supplementary Information

**Additional file 1.** SPIRIT Statement check list.

## Data Availability

All individual participant data and materials collected during the trial will be shared after deidentification. Will make the database available upon reasonable request. The data and material-sharing time frame will begin 3 months after trial completion and end 11 months following trial completion. During the mentioned time frame, data will be shared with anyone who wishes to access it for any type of analysis or purpose. The time planned for this study is 24 months, which is further divided into two major phases. The 1st phase is the preparation of the study tools and materials. The second phase is applying the trial methods mentioned above. The expected date of trial initiation is June 20th 2020. Further details of the study plan are mentioned in the table below (Tables 1 and 2):

## Abbreviations

PTL: Preterm labor
BMI: Body mass index
OR: Odd ratios
CI: Confidence interval
